# Effects of Chronic Thermal Stress on Performance, Energy Metabolism, Antioxidant Activity, Brain Serotonin, and Blood Biochemical Indices of Broiler Chickens

**DOI:** 10.3390/ani11092554

**Published:** 2021-08-31

**Authors:** Omar A. Ahmed-Farid, Ayman S. Salah, Mohamed Abdo Nassan, Mahmoud S. El-Tarabany

**Affiliations:** 1 Physiology Department, National Organization for Drug Control and Research (NODCAR), Giza 35521, Egypt; ebntaimya@yahoo.com; 2 Department of Animal Nutrition and Clinical Nutrition, Faculty of Veterinary Medicine, New Valley University, El-Kharga 72511, Egypt; asabry3999@yahoo.com; 3 Department of Clinical Laboratory Sciences, Turabah University College, Taif University, Taif 21944, Saudi Arabia; m.nassan@tu.edu.sa; 4 Department of Animal Wealth Development, Faculty of Veterinary Medicine, Zagazig University, Zagazig 44511, Egypt

**Keywords:** broiler, performance, heat stress, metabolism, energy

## Abstract

**Simple Summary:**

In the tropical and subtropical regions, heat stress is the main limiting factor of poultry industries. In this context, broilers are more liable to thermal stress due to their fast growth, rapid metabolic rate, and high level of production. The aim of the current work was to analyze changes in the brain serotonin, energy metabolism, antioxidant biomarkers, and blood chemistry of broiler chickens subjected to chronic thermal stress. Thermal stress disturbed the antioxidant defense system and energy metabolism and exhausted ATP levels in the liver tissues of broiler chickens. Interestingly, chronic thermal stress reduced the level of brain serotonin and the activity of CoQ10 in liver tissues.

**Abstract:**

The aim of this paper was to investigate the effects of chronic thermal stress on the performance, energy metabolism, liver CoQ10, brain serotonin, and blood parameters of broiler chickens. In total, 100 one-day-old chicks were divided into two equal groups of five replicates. At 22 days of age and thereafter, the first group (TN) was maintained at a thermoneutral condition (23 ± 1 °C), while the second group (TS) was subjected to 8 h of thermal stress (34 °C). The heat-stressed group showed significantly lower ADFI but higher FCR than the thermoneutral group (*p* = 0.030 and 0.041, respectively). The TS group showed significantly higher serum cholesterol, ALT, and AST (*p =* 0.033, 0.024, and 0.010, respectively). Meanwhile, the TS group showed lower serum total proteins, albumin, globulin, and Na+ than the TN group (*p* = 0.001, 0.025, 0.032, and 0.002, respectively). Furthermore, the TS group showed significantly lower SOD and catalase in heart tissues (*p* = 0.005 and 0.001, respectively). The TS group showed significantly lower liver ATP than the TN group (*p* = 0.005). Meanwhile, chronic thermal stress significantly increased the levels of ADP and AMP in the liver tissues of broiler chickens (*p* = 0.004 and 0.029, respectively). The TS group showed significantly lower brain serotonin (*p* = 0.004) and liver CoQ10 (*p* = 0.001) than the TN group. It could be concluded that thermal stress disturbed the antioxidant defense system and energy metabolism and exhausted ATP levels in the liver tissues of broiler chickens. Interestingly, chronic thermal stress reduced the level of brain serotonin and the activity of CoQ10 in liver tissues.

## 1. Introduction

For decades, thermal stress has been considered the main limiting factor of the poultry industry in tropical and subtropical regions [1]. Indeed, increased global warming will exaggerate heat stress-related problems [2]. When housing temperatures surpass comfort level, birds try to minimize metabolic heat production through a reduction in feed consumption [3], which consequently reduces growth rate and profitability [4]. In this context, broiler chickens are more susceptible to thermal stress due to their high growth rate and related high metabolic heat production [5].

Several parameters are traditionally measured as thermal stress biomarkers in broiler chickens, including plasma concentrations of corticosterone, cholesterol, protein fractions, electrolytes, and antioxidants [6]. Furthermore, thermal stress adversely affects cellular organelles, impairing oxidative metabolism and the functional structures of membranes [7]. Thermal stress also increases plasma cholesterol, reduces serum proteins, activates the lipid peroxidation process in blood and tissues, and disturbs electrolyte balance in the body [8]. Indeed, heat stress can increase the production of ROS in mitochondria and, consequently, reduce energy generation efficiency and adenosine triphosphate (ATP) synthesis [9]. Mujahid et al. [10] also stated that mitochondria are more susceptible to oxidative damage, probably due to the high percentage of polyunsaturated fatty acids and proteins in their membranes. Harmful heavy metal cadmium can cause mitochondrial damage and oxidative stress in common carp (*Cyprinus carpio* L.) gills [11]. Additionally, the toxic gas ammonia can damage mitochondria, affect energy metabolism, and lead to oxidative stress in the chicken thymus [12]. Coenzyme Q (CoQ) acts as an electron carrier and has been considered a major oil-soluble antioxidant within cellular membranes and other lipophilic structures. Furthermore, its antioxidant properties include the inhibition of the oxidative process of lipids and proteins, as well as the regeneration of other antioxidants such as vitamin E [9]. Meanwhile, studies on the bioavailability of CoQ under thermal stress conditions are relatively limited.

Serotonin (5-HT) is an important signaling molecule involved in several neurotransmitter functions of the brain. Outside the brain, serotonin plays a key role in regulating the contractility of the gastrointestinal smooth muscle and epithelial secretions [13]. In vertebrates, serotonin is stored in the enterochromaffin cells. These cells are numerous in the mucosal epithelium of the gastrointestinal tract [14]. Furthermore, it is believed that serotonin regulates the process of bone formation, as well as the mechanism of bone resorption [15]. In this context, Calefi et al. [16] reported that acute stress conditions such as environmental temperature increases the concentration of serotonin in brain tissues; however, chronic stress conditions decrease the level of brain serotonin. Therefore, the aim of the current work is to analyze changes in the brain serotonin, liver CoQ10, energy metabolism, antioxidant biomarkers, and blood chemistry of broiler chickens subjected to chronic thermal stress.

## 2. Materials and Methods

### 2.1. Birds and Management

In total, 100 one-day-old chicks (Ross) were divided into 2 equal groups of five replicates (10 birds/replicate). Housing pens were provided with fresh wood shavings (15 birds/m^2^), and birds had free access to feed and water. Regular supplementation of heat was performed by digital heaters to maintain a stable housing temperature (automated diesel heater; Naganpuriya High Tech Farming Equipment). Both groups were housed at 34 °C during the first week of age. Thereafter, the temperature was reduced gradually to reach 23 °C at 21 days of age. The first group (TN) was maintained at a thermoneutral condition (23 ± 1 °C), while the other group (TS) was subjected to 8 h of heat stress at 34 °C (08:00–16:00 and 23 ± 1 °C for the remaining time). The relative humidity was adjusted to 58 ± 3%, and regular observation was practiced to check the stability of the housing temperature and ventilation. Furthermore, the mortality rate was recorded in both experimental groups. A routine vaccination program against Newcastle disease and Gumboro disease was applied. All birds fed the same starter and grower–finisher diets [17] (see Table 1). Body weight and feed intake were recorded weekly to determine the average daily feed intake (ADFI) and feed conversion ratio (FCR) during the period of 22–42 days of age.

### 2.2. Blood Sampling and Biochemical Analyses

At 42 days of age, 3 mL blood samples were collected in plain tubes (10 birds/group). The serum samples were separated (1200× *g*) and stored at −20 °C. The concentrations of serum cholesterol, AST, ALT, total protein, and albumin were determined by Roch diagnostics kits (GmbH, Mannheim, Germany). The activity of total antioxidant capacity (TAC) in the serum samples was measured (Cell Biolabs kits, Inc., San Diego, CA, USA). The levels of serum sodium (Na) and potassium (K) were estimated by an electrolyte analyzer (Shenzhen Kindle Medical Devices Co. Ltd., Shenzhen, China).

### 2.3. Determination of Brain Serotonin

At 42 days of age, two birds from each replicate (10 birds/group) were fasted for 6 h and slaughtered according to the Islamic method (HALAL Slaughter) of the Malaysian institutes [18]. The main jugulars of broiler chickens are severed with sharp knives without using any anesthetics to get effective bleeding. Brain samples (striatum, frontal cortex, and hypothalamus) were homogenized in HPLC-grade methanol solution [19]. After the derivatization process was completed, the dried samples were mixed with a diluent composed of 0.71 g of disodium hydrogen phosphate (pH of 7.4) plus 5% acetonitrile. The homogenate of each sample was run at (4000 rpm) for 10 min, and the supernatant was collected. The level of brain serotonin was determined by HPLC in accordance with the method described by Pagel et al. [20]. Compared with the standard, the resulting chromatogram had to characterize the concentration of serotonin as μg per gram of brain tissue.

### 2.4. Determination of Antioxidant Activity in Heart Tissues

Five heart samples were obtained from each group. Each homogenate heart sample was prepared in a 10 mM phosphate buffer (pH 7.4). Then, the suspension was centrifuged at 12,000× *g* for 10 min at 4 °C to collect the clear supernatant. The activities of superoxide dismutase (SOD) and catalase (CAT) in the supernatant were determined according to the method described by Ahmed-Farid et al. [21]. The SOD activity was observed at two-minute intervals. The activity was illustrated as the amount of enzyme that inhibits the autoxidation of pyrogallol. Based on the decomposition of H_2_O_2_, the CAT activity was measured [22].

### 2.5. Determination of CoQ10 and Energy Biomarkers in Liver Tissues

Liver adenosine contents of tri-, di-, and monophosphate (ATP, ADP, and AMP) were quantified by HPLC in accordance with the protocol described by Teerlink et al. [23]. The serum samples were prepared by mixing 0.2 mL of serum with 0.3 mL of methanol (70%), then centrifuged (5000 rpm) at 4 °C for 20 min to obtain the supernatant. Fifty hundred microliters of liver tissue were homogenized with ice-cold 10% potassium chloride, then centrifuged at 5000 rpm for 20 min to collect the clear supernatant. For deprotonization, 200 µL of supernatant was mixed with 1 mL of methanol (70%) and prepared for HPLC analysis (Nova-PakTM C18 column). The reports and chromatograms were obtained from the ChemStation program, with a wavelength of 254 nm and an injection volume of 20 µL. Liver CoQ10 contents were quantified by HPLC (Agilent HP 1200 series apparatus, Santa Clara, CA, USA) according to the modified protocol of Niklowitz et al. [24].

### 2.6. Statistical Analysis

The data were analyzed using ANOVA procedures in the IBM SPSS software program (Version 16.0; IBM Corp., New York, NY, USA). For performance traits, each pen was considered an experimental unit. The model included the fixed effects of the thermal treatment (two levels: TN and TS) and the random effect of experimental error. Body weight at 21 days of age was included as a covariate in the statistical model. The outputs are expressed as means and the standard error of means (SEM). The results are considered significant at level *p* ˂ 0.05.

## 3. Results

**Figure 1:**
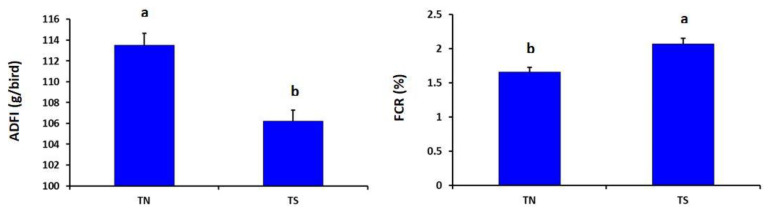
Effects of chronic thermal stress on average daily feed intake (ADFI) and feed conversion ratio (FCR) of broiler chickens (*p* = 0.030 and 0.041, respectively). TN: Thermoneutral group; TS: Thermal-stressed group. ^a,b^ Values with different superscripts differ significantly.

As described in Figure 1, the heat-stressed group showed significantly lower ADFI but higher FCR than the thermoneutral group (*p* = 0.030 and 0.041, respectively).

The effects of chronic thermal stress on the blood chemistry of broiler chicken are illustrated in Table 2. The TS group showed significantly higher serum cholesterol, ALT, and AST than the TN group (*p* = 0.033, 0.024, and 0.010, respectively). Meanwhile, the TS group showed significantly lower serum total proteins, albumin, globulin, and Na^+^ (*p* = 0.001, 0.025, 0.032, and 0.002, respectively). The levels of serum K^+^ and A/G ratio did not differ between experimental groups (*p* = 0.603 and 0.239, respectively).

The effects of chronic thermal stress on the antioxidant activity of broiler chickens are illustrated in Table 3. Thermal stress significantly decreased serum TAC in broilers compared with broilers maintained at thermoneutral conditions (*p* = 0.021). Furthermore, the TS group showed significantly lower SOD and catalase in heart tissues (*p* = 0.005 and 0.001, respectively).

The effects of thermal stress on energy biomarkers in liver tissues are illustrated in Table 4. The TS group showed significantly lower liver ATP than the TN group (*p* = 0.005). Meanwhile, chronic thermal stress significantly increased the levels of ADP and AMP in the liver tissues of broiler chickens (*p* = 0.004 and 0.029, respectively). The level of liver Na,K-ATPase did not differ between experimental groups (*p* = 0.115).

**Figure 2:**
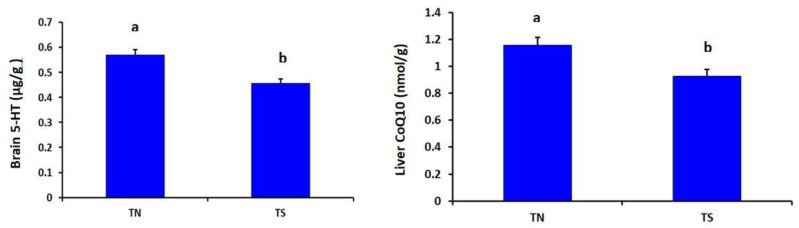
Effects of chronic thermal stress on brain serotonin and liver CoQ10 of broiler chickens (*p* = 0.004 and 0.001, respectively). TN: Thermoneutral group; TS: Thermal-stressed group. ^a,b^ Values with different superscripts differ significantly.

The TS group showed significantly lower brain serotonin (*p* = 0.004) and liver CoQ10 (*p* = 0.001) than the TN group (Figure 2).

## 4. Discussion

Adverse environmental conditions such as ambient temperature may cause significant economic losses to the poultry industry due to their negative effects on performance indices. In this context, broiler chickens are more susceptible to stress conditions, probably due to their rapid growth rate [25]. The majority of the literature, however, has focused on acute or short-duration thermal stresses. Herein, chronic thermal stress conditions reduced feed intake and increased the FCR in broiler chickens. Consistent with our findings, Roushdy et al. [7] stated that chronic thermal stress (daily, 6 h at 34 °C, for three consecutive weeks) deteriorates the FCR in both Ross and Cobb broiler chickens. During the late fattening period, Quinteiro-Filho et al. [4] also reported a significant reduction in body gain and an increase in the FCR when broilers were exposed to 36 °C for 10 h daily. The adverse effects of thermal stress on broiler performance may be attributed to the quick synthesis and release of cortisol in the adrenal cortex. Additionally, stress conditions may impair intestinal integrity, causing an inability to absorb nutrients [26,27]. Unsurprisingly, the reduced feed consumption in the TS group is consistent with previous studies [28,29,30]. Attia and Hassan [31] supposed that thermal stress stimulates the peripheral thermal receptors of broiler chickens to transmit inhibitory nerve impulses to the appetite center in the hypothalamus and, consequently, reduce feed consumption. Others suggested that brain monoamines, including serotonin, are involved in the control of feed intake [32]; hence, stress conditions may activate the central monoaminergic system [33]. Additionally, serotonin is an essential messenger to modulate the brain–gut connection, as well as the maintenance of gastrointestinal motility and visceral sensation [34]. In this context, the lower concentration of brain serotonin in the heat-stressed group may explain the reduction in feed intake in broiler chickens.

Blood biochemical indexes can indicate the metabolic and physiological responses of broiler chickens to different nutritional and environmental conditions [35]. In the current study, the TS group showed higher concentrations of serum ALT and AST. This may indicate some sort of hepatic damage in heat-stressed broilers [36]. Consistent with these findings, Zhang et al. [37] reported that exposure to thermal stress (34 ± 1 °C for 8 h) increases the activities of serum AST and ALT. Moreover, Lan et al. [38] reported that high ambient temperature induces liver damage and increases serum AST and ALT. Herein, chronic thermal stress deteriorated the indices of serum proteins, as well as increased the level of serum cholesterol. Similarly, Attia et al. [39] reported a significant increase in serum cholesterol when broilers were subjected to prolonged thermal stress (36 °C). Others recorded similar cholesterol findings when birds were exposed to cyclic thermal stress conditions [40]. Thermal stress disturbs the electrolyte balance in broiler chickens. Consistent with these findings, Zaglool et al. [41] reported that serum Na+ was significantly reduced when broiler chickens were subjected to 6 h of thermal stress. This electrolyte disturbance may be attributed to increased water consumption and the related hemodilution process [42], or to the loss of body water due to decreased extracellular fluids [43].

Thermal stress accelerates the lipid peroxidation process and, consequently, exhausts the antioxidant defense system in broiler chickens [44]. The current study also demonstrated a significant reduction in the level of serum TAC of heat-stressed broilers. Xue et al. [45] recorded a great depletion in serum antioxidant activity when Arbor Acres broiler chickens were exposed to cyclic thermal stress. Under thermal stress conditions, the expected exhaustion of the antioxidant defense system in broiler chickens may be due to the rapid oxidation process and related cellular damages [46]. It is believed that both catalase and SOD act as the first line of the antioxidant defense system in the body tissues. Herein, chronic thermal stress reduced the levels of SOD and catalase in the heart tissues of broiler chickens. Consistent with these findings, Zeng et al. [47] reported that the activities of SOD and catalase were significantly decreased in liver tissues when Pekin ducks were subjected to short-term heat stress (*p* < 0.05).

The accumulation of ROS in mitochondria is associated with thermal stress, as well as with subsequent damage to proteins, lipids, and DNA structures. When thermal stress is prolonged, mitochondrial homeostasis is disturbed, and ATP synthesis is decreased [9]. In the present study, chronic thermal stress reduced the concentration of ATP in liver tissues. Additionally, the increased liver ADP in heat-stressed broilers indicates the impaired energy metabolism of birds. In this context, Joung et al. [48] suggested that stress-induced depletion of ATP in liver tissues induces necrosis patterns with more severe liver injuries. Of note, it is believed that short-term stress disturbs the activity of Na^+^,K^+^-ATPase in broiler chickens [49]. In the current study, chronic thermal stress did not exert significant changes in the activity of Na^+^,K^+^-ATPase in liver tissues. However, Chen et al. [50] demonstrated that acute heat stress significantly reduced Na^+^-K^+^-ATPase in the intestinal mucosa of chickens. Coenzyme Q10 is a naturally occurring lipophilic compound that regulates the process of oxidative phosphorylation in mitochondria and acts as an antioxidant [51]. It is also involved in the synthesis of ATP and regulation of the bioenergetics pathway [52]. In the current trial, chronic thermal stress reduced the activity of CoQ10 in the liver tissues of broiler chickens. In this context, Xu et al. [53] reported that CoQ10 reduces oxidative damage to chicken myocardial cells.

Serotonin is a neurotransmitter involved in different neurological functions of the brain and nervous system. It is also an important messenger in the digestive tract and in the regulation of gastrointestinal motility and visceral sensation [34]. In the current study, the heat-stressed group showed significantly lower brain serotonin than the thermoneutral group. In this context, the reduced FI in the heat-stressed group may be attributed to fluctuating serotonin levels. Supporting this explanation, Raybould [13] suggested that serotonin plays a crucial role in controlling the contractility of smooth muscle in the GIT, as well as the activity of secretory epithelial cells. Consistent with our findings, Buraczewska et al. [54] stated that the addition of crystalline tryptophan to broiler diets increased the concentration of serotonin in brain tissues, with a significant increase in feed consumption. On the contrary, Denbow et al. [55] reported that tryptophan supplements increased the level of brain serotonin in turkeys but had no effect on feed intake.

## 5. Conclusions

It could be concluded that chronic thermal stress reduces the performance, serum proteins, and TAC, as well as the activity of SOD and catalase in the heart tissues of broiler chickens. Furthermore, chronic thermal stress disturbs energy metabolism by exhausting ATP levels in the liver tissues of broilers. Interestingly, chronic thermal stress reduced the level of brain serotonin and activity of CoQ10 in the liver tissues in broiler chickens. The present results may be helpful to target appropriate strategies to minimize the adverse effects of thermal stress in broiler chickens.

## Figures and Tables

**Table 1:** Ingredient composition and calculated chemical analysis of the basal diets.

Ingredients	Starter Period (1–21 d, g kg^−1^)	Grower–Finisher Period (22–42 d, g kg^−1^)
Yellow maize	605.0	650.0
Soybean meal (48%)	308.0	250.0
Corn gluten (60%)	40.0	35.0
Maize oil	-	18.0
Di-calcium phosphate	23.0	23.0
Limestone	14.0	14.0
DL-methionine	1.0	1.0
Lysine	1.0	1.0
Vitamin and trace mineral mix	3.5	3.5
Salt (NaCl)	3.5	3.5
Coccidostate	1.0	1.0
Calculated analysis		
^1^ ME (KJ/kg)	12342	12949
Crude protein	224.0	197.5
Calcium	10.5	10.5
Available phosphorus	4.5	4.5
Lysine	11.8	11.4
Methionine	4.8	4.5

^1^ ME: metabolizable energy.

**Table 2:** Effect of chronic thermal stress on blood chemistry of broiler chickens.

Parameter	Experimental Groups			
	TN ^1^	TS ^2^	SEM ^3^	*p*-Value
Total protein (g/dL)	6.63	5.86	0.14	0.001
Albumin (g/dL)	4.38	4.07	0.07	0.025
Globulin (g/dL)	2.25	1.79	0.11	0.032
Albumin/Globulin ratio	2.01	2.32	0.13	0.239
Cholesterol (mg/dL)	79.33	84.95	1.37	0.033
^4^ ALT (U/L)	54.96	60.60	1.31	0.024
^5^ AST (U/L)	44.40	53.35	1.91	0.010
K^+^ (mmol/L)	4.04	3.94	0.08	0.603
Na^+^ (mmol/L)	133.4	113.9	3.69	0.002

^1^ Thermoneutral group; ^2^ thermal-stressed group; ^3^ standard error of means; ^4^ alanine aminotransferase; ^5^ aspartate aminotransferase.

**Table 3:** Effect of chronic thermal stress on antioxidant activity of broiler chickens.

Parameter	Experimental Groups			
	TN ^1^	TS ^2^	SEM ^3^	*p*-Value
^4^ SOD (U/g, heart)	43.20	35.21	1.60	0.005
^5^ Catalase (U/g, heart)	12.78	9.22	0.57	0.001
^5^ TAC (U/L, serum)	1.51	1.38	0.03	0.021

^1^ Thermoneutral group; ^2^ thermal-stressed group; ^3^ standard error of means; ^4^ superoxide dismutase; ^5^ total antioxidant capacity.

**Table 4:** Effect of chronic thermal stress on energy biomarkers in liver tissues of broiler chickens.

Parameter	Experimental Groups			
	TN ^1^	TS ^2^	SEM ^3^	*p*-Value
^4^ ATP (μg/g)	36.86	30.15	1.34	0.005
^5^ ADP (μg/g)	22.63	26.11	0.68	0.004
^6^ AMP (μg/g)	9.37	10.38	0.25	0.029
Na,K-ATPase (μmol/g)	469.9	515.2	13.5	0.115

^1^ Thermoneutral group; ^2^ thermal-stressed group; ^3^ standard error of means; ^4^ adenosine triphosphate; ^5^ adenosine diphosphate; ^6^ adenosine monophosphate.

## Data Availability

Data sharing is not applicable. All data analyzed during this study are included in this published paper.
